# ST6GAL1 Is a Functional Regulator of UVA-Induced Photoaging in Human Dermal Fibroblasts

**DOI:** 10.3390/cells15161497

**Published:** 2026-08-20

**Authors:** Jiangming Zhong, Ling Liang, Man Wu, Yuting Liang, Menggeng Li, Cheuk-Lun Lee, Peng Shu

**Affiliations:** 1 HBN Research Institute and Biological Laboratory, Shenzhen Hujia Technology Co., Ltd., Shenzhen 518000, China; 2 Guangdong Engineering Technology Research Center for Functional Skincare Innovation, Shenzhen Hujia Technology Co., Ltd., Shenzhen 518000, China; 3 Department of Health Technology and Informatics, The Hong Kong Polytechnic University, Kowloon, Hong Kong SAR, China

**Keywords:** photoaging, glycosylation, ST6GAL1, human dermal fibroblast, α2,6-sialylation, cellular senescence, UVA

## Abstract

Skin photoaging, primarily driven by UVA radiation, is characterized by the accumulation of senescent fibroblasts and the degradation of the extracellular matrix (ECM). While the roles of reactive oxygen species (ROS) and matrix metalloproteinases (MMPs) are well-documented, the regulatory impact of post-translational glycosylation in this process remains poorly understood. We established a UVA-induced photoaging model in human dermal fibroblasts (HDFs) and employed bulk mRNA-seq and high-throughput lectin microarrays to profile glycomic alterations. The functional role of the sialyltransferase ST6GAL1 was investigated through pharmacological inhibition of cellular sialylation (3Fax-Neu5Ac), siRNA-mediated knockdown, and gain-of-function overexpression. Mechanistic insights were gained via RAS-ERK pathway analysis and validated in a 3D reconstructed human full-thickness skin model (T-Skin™). Glycomic profiling revealed that UVA irradiation triggers a broad increase in α2,6-sialylation in HDFs. We identified ST6GAL1 as the primary enzymatic driver of this remodeling, with its expression upregulated in both photoaged HDFs and 3D skin models. Functional assays demonstrated that ST6GAL1 overexpression induces hallmark features of photoaging, including p16, MMP and γ-H2AX upregulation, G0/G1 cell cycle arrest and increased SA-β-gal activity. Conversely, pharmacological or genetic inhibition of ST6GAL1 effectively mitigated the photoaged phenotype. Mechanistically, ST6GAL1 regulates the expression of p16 via the activation of the RAS-ERK signaling cascade. Our study identifies ST6GAL1-mediated α2,6-sialylation as a novel functional hallmark of skin photoaging, highlighting the association of ST6GAL1 with the RAS-ERK-p16 axis as a potential regulator for targeting UVA-induced skin photoaging and dermal senescence.

## 1. Introduction

Skin photoaging, driven primarily by cumulative ultraviolet (UV) exposure, represents a major extrinsic aging process with profound implications for global aging populations [1]. UVA radiation (320–400 nm), due to its deeper dermal penetration capacity, is recognized as the primary driver of photoaging, directly contributing to wrinkle formation and a loss of skin elasticity [2]. Beyond its visible effects on skin appearance, photoaging fundamentally disrupts dermal homeostasis, leading to persistent low-grade inflammation and increased susceptibility to neoplastic changes [3]. Given the escalating global UV exposure due to both environmental changes and lifestyle factors, elucidating the molecular mechanisms underlying photoaging has emerged as a critical priority in dermatological research.

The current understanding of the molecular mechanism of skin photoaging is that it is mechanistically driven by UVA-induced reactive oxygen species (ROS), which stimulate dermal fibroblasts to overexpress matrix metalloproteinases 1 and 3 (MMP-1,3) and accelerate the degradation of type I and III collagen [2,4]. Recent emerging research has also highlighted the pivotal involvement of cellular senescence in skin photoaging, offering new perspectives on its pathological mechanisms [5]. Senescent cells exhibit functional impairment, cell cycle arrest, and the secretion of diverse factors collectively termed the senescence-associated secretory phenotype (SASP) [6]. These senescent cells can be identified in both dermal and epidermal layers through characteristic biomarkers including SA-β-galactosidase activity, elevated expression of cyclin-dependent kinase inhibitors (CDKN2A/p16 and CDKN1A/p21), and DNA damage markers (γ-H2AX) [7,8]. Photoaging also contributes to the accumulation of glycation stress in skin by the formation of advanced glycation end products (AGEs) [9]. However, the role of post-translational modifications, particularly glycosylation as the most abundant, diverse, and complex form of post-translational modification, remains underexplored in dermal fibroblast photoaging. Protein glycosylation involves the addition or removal of oligosaccharides (glycans) by glycosyltransferases and glycosidases [10]. The structural diversity of glycans plays a critical role in modulating glycoprotein conformation, stability, solubility and binding interactions, which further elicits downstream signaling cascades that regulate fundamental physiological and pathological processes including tumorigenesis [11], inflammation [12] and aging [13].

Recently, studies have revealed alterations in cell surface glycans on fibroblasts during aging and cellular senescence. For example, in epidermal stem cell aging, the upregulation of sialylation and downregulation of mannosylation were also observed [14]. A study employing lectin microarrays also identified senescence-associated changes in the global membrane glycosylation patterns of glycoproteins in human skin fibroblasts [15]. Nevertheless, the functional implications of these glycosylation changes on fibroblast behavior and senescence, particularly under photoaging conditions, remain poorly understood.

In this study, we hypothesized that UVA exposure triggers a specific cellular glyco-remodeling event, and that the sialyltransferase ST6GAL1 acts as an enzymatic driver of dermal photoaging by regulating fibroblast senescence. To test this hypothesis, we established a UVA-induced photoaging model in human dermal fibroblasts (HDFs) and employed lectin microarray analysis to profile alterations in glycosylation patterns. Our results revealed that sialylation represents a critically dysregulated pathway and identified ST6GAL1 as a key functional mediator driving photoaging progression via modulation of various aspects of cellular senescence, including the senescence-associated secretory phenotype (SASP), SA-β-gal activity, and cell cycle arrest. Overall, this work establishes ST6GAL1-mediated sialylation as a novel regulatory axis in human skin dermal senescence.

## 2. Method and Materials

### 2.1. Cell Culture and UVA Radiation

Human dermal fibroblasts (HDFs), obtained from the Procell System (CP-H103, Procell, Wuhan, China), were seeded into 6-well plates and maintained in Dulbecco’s Modified Eagle’s Medium (DMEM) containing 10% fetal bovine serum (FBS) and 1% penicillin–streptomycin (Life Technologies, CA, USA) at 37 °C in a humidified 5% CO_2_ atmosphere. Prior to UVA irradiation, the cells were washed and covered with phosphate-buffered saline (PBS). Irradiation was performed using a UVA crosslinker (UCL-3500L, Luyor, Shanghai, China) at a dose of 5 J/cm^2^ (365 nm) for 300 s. Following exposure, PBS was replaced with fresh culture medium. This irradiation procedure was repeated daily for three consecutive days. Control groups were handled identically but were shielded from UVA exposure.

### 2.2. Western Blot Analysis

Total cellular proteins were extracted using RIPA buffer (P0025, Beyotime, Shanghai, China) supplemented with phosphatase inhibitor cocktail II (HY-K0022, MedChemExpress, NJ, USA) and protease inhibitor cocktail (HY-K0010, MedChemExpress, NJ, USA). The protein extracts were denatured with 5× SDS loading buffer (P0015L, Beyotime, Shanghai, China), separated by SDS-PAGE, and transferred onto PVDF membranes (IPVH00010, Millipore, CA, USA). The membranes were then blocked with QuickBlock™ Blocking Buffer for Western blot analysis (P0252, Beyotime, Shanghai, China) and subsequently incubated with the following primary antibodies: COL1 (1:1000, 14695-1-AP Proteintech, Chicago, IL, USA); p16 (1:1000, 10883-1-AP Proteintech, Chicago, IL, USA); p21 (1:1000, ab109520, Abcam, UK); γ-H2A (1:1000, Abcam, ab229914, UK); β-actin (1:5000, 20536-1-AP, Proteintech, Chicago, IL, USA); FUT1 (1:1000, 17956-1-AP, Proteintech, Chicago, USA); FUT2 (1:1000, 20288-1-AP, Proteintech, Chicago, IL, USA); ST6GAL1 (1:1000, 68633-1-Ig, Proteintech, Chicago, IL, USA); MMP1 (1:1000, 10371-2-AP, Proteintech, Chicago, IL, USA); MMP3 (1:1000, 17873-1-AP, Proteintech, Chicago, IL, USA); ERK (1:1000, 66192-1-Ig, Proteintech, Chicago, IL, USA); ERK (phospho Thr202/Tyr204) (1:1000, 28733-1-AP, Proteintech, Chicago, IL, USA); and RAS (1:1000, 60309-1-Ig, Proteintech, Chicago, IL, USA). Horseradish peroxidase (HRP)-conjugated goat anti-rabbit and anti-mouse secondary antibodies were also applied (1:5000, 111-035-003 and 115-035-003, Jackson, CA, USA). Immunoreactive bands were visualized with BeyoECL SuperMoon (P0018HM, Beyotime, Shanghai, China) and documented using an eBlot imaging system (Genscript, Jiangsu, China). Quantitative analysis of protein expression was carried out by measuring the gray density of the bands with ImageJ 1.51 software (NIH, Bethesda, MD, USA).

### 2.3. Senescence-Associated β-Galactosidase Staining

Senescence-associated β-galactosidase (SA-β-gal) activity in treated HDFs was assessed using a β-Galactosidase Staining Kit (C0602, Beyotime, Shanghai, China) in accordance with the manufacturer’s instructions. The experimental procedure was conducted as follows: cells were initially rinsed with PBS and subsequently fixed by incubation with 1 mL of β-galactosidase fixative solution for 15 min at ambient temperature. Following fixation, residual fixative was eliminated through three successive washes with PBS, each lasting 3 min. After complete removal of the wash buffer, each well was supplemented with 2 mL of freshly prepared staining working solution. The specimens were then incubated overnight at 37 °C prior to microscopic examination (DMi8, Leica, Wetzlar, Germany).

### 2.4. Total RNA Extraction and Quantitative PCR (qPCR) Analysis

Total RNA was extracted using the TransZol Up Plus RNA Kit (ER501-01-V2, Transgen, Beijing, China). Complementary DNA (cDNA) was then synthesized from the extracted RNA using the 5X Evo M-MLV RT Reaction Mix (AG11728, Agbio, Changsha, China). The resulting cDNA was subsequently used as a template for quantitative polymerase chain reaction (qPCR) analysis, which was performed with SYBR Mix (11202ES, Yeasen, Shanghai, China). The specific primer sequences used for qPCR are listed in Table 1.

### 2.5. Bulk mRNA Sequencing

Bulk mRNA sequencing was performed on HDF cells with three biological replicates under the treatment conditions described previously. Total RNA was isolated using TRIzol™ Reagent (15596026CN, Gibco, Waltham, MA, USA) following the manufacturer’s instructions. Eukaryotic mRNA was enriched and purified using VAHTS mRNA capture beads conjugated with Oligo(dT) primers. The purified RNA was then fragmented into 250–450 bp pieces and subsequently converted into a cDNA library. Library quality was assessed using Qubit 4.0, LabChip or Fragment Analyzer (FA) systems, along with ABI QuantStudio 12K real-time PCR. High-throughput sequencing was performed on the Illumina Novaseq™ 6000 platform, yielding ~6 Gb of raw data per sample. Adapter trimming and quality filtering were conducted with fastp (v0.23.2), followed by rRNA removal using SortMeRNA (v4.3.4). Clean reads were aligned to the GRCh38 human reference genome using STAR (v2.7.10a), achieving a total mapping rate >99%. Differential expression analysis was performed with DESeq2 (v1.34.0), and genes with |log2 fold change| > 1 and p.adjust < 0.05 were considered significantly differentially expressed. GSEA was conducted using the gseGO function from clusterProfiler (v4.18.4) in R (v4.4.2). All genes with baseMean > 0 obtained from DESeq2 analysis were included as inputs. Genes were ranked by the log2 fold change derived from DESeq2, with the sign indicating the direction of regulation. Gene Ontology gene sets were retrieved from org.Hs.eg.db (v3.20.0). Gene sets with fewer than 10 or more than 500 genes were excluded. Significantly enriched gene sets were defined as those with p.adjust < 0.05.

### 2.6. Lectin Microarray Analysis

Lectin profiling was performed using the Lectin Array 70 kit (Abcam, ab272786) following the manufacturer’s instructions. Protein samples (30 μg) were labeled with biotin using the provided Labeling Reagent, followed by dialysis against PBS (pH 8.0) at 4 °C. The array slides were air-dried for 1–2 h, blocked with Sample Diluent for 30 min, and incubated with biotin-labeled samples (100 μL/well) at room temperature for 2 h. After washing with Wash Buffers I and II, the slides were incubated with Cy3-streptavidin for 1 h in the dark. Following final washes, slides were dried by centrifugation and scanned at 570 nm using a LuxScan™ 10K microarray scanner (CapitialBio, Beijing, China). The map of Lectin Array 70 is presented in Supplementary Figure S1. Signal intensities were extracted and normalized to positive controls.

### 2.7. FACS Analysis of Lectin Binding

Culture medium was removed, and cells were washed with PBS. Cells were harvested by trypsinization, washed with PBS, and centrifuged at 500× *g* for 5 min at 4 °C. The pellet was resuspended in ice-cold PBS containing 2% BSA, and cell number was determined. The cell suspension was adjusted to 0.1–1 × 10^6^ cells/mL, and 100 µL aliquots were transferred to tubes. Blocking was performed using Carbo-free blocking solution (SP-5040-125, Vector Laboratories, Newark, CA, USA). A master mix was prepared in FACS buffer containing the following fluorescein-labeled lectins at an initial concentration of 1 µg/mL at 4 °C: Concanavalin A (Con A) (FL-1001-25,Vector Laboratories, Newark, CA, USA), Peanut Agglutinin (PNA) (FL-1071-5, Vector Laboratories, Newark, CA, USA), Dolichos Biflorus Agglutinin (DBA) (FL-1031-2, Vector Laboratories, Newark, CA, USA), Ulex europaeus agglutinin I (UEA I) (FL-1061-2,Vector Laboratories, Newark, CA, USA), Sambucus nigra lectin (SNA, EBL) (FL-1301-2, Vector Laboratories, Newark, CA, USA), and RCA120 (FL-1081-1, Vector Laboratories, Newark, CA, USA). Cells were resuspended in 50–100 µL of master mix and incubated on ice or at 4 °C for 30 min in the dark. After incubation, cells were washed twice with 200 µL of FACS buffer or cold PBS. Finally, cells were resuspended in 300 µL of FACS to obtain a single-cell suspension prior to FACS analysis.

### 2.8. Cell Cycle Analysis

Cell cycle analysis was performed using the Cell Cycle Assay Kit (Red Fluorescence) (E-CK-A351, Elabscience, Houston, TX, USA). Briefly, 5 × 10^5^ cells were harvested, washed with 1 mL PBS, and fixed by overnight incubation in 70% (*v*/*v*) ethanol at −20 °C. After fixation, cells were washed with PBS for 15 min at room temperature and then treated with RNase A solution at 37 °C for 30 min. Cellular DNA was stained with propidium iodide (PI) at a concentration of 50 µg/mL for 30 min at 4 °C in the dark. Flow cytometric analysis was conducted using a Beckman CytoFlex flow cytometer (Indianapolis, IN, USA), and cell cycle distribution was evaluated with ModFit LT 6.0 software (Cytonome Verity, Lexington, MA, USA).

### 2.9. Construction of ST6GAL1 Overexpression Vector and Transfection

An overexpression vector was generated using the pcDNA3.1(+) plasmid and introduced into cells via Lipofectamine™ 3000 reagent (L3000015, Invitrogen, Carlsbad, CA, USA). In brief, cells were plated to achieve 70–90% confluence at the time of transfection. Lipofectamine™ 3000 reagent was diluted in Opti-MEM Medium (31985070, Gibco, Waltham, MA, USA) and mixed. Separately, DNA was diluted in Opti-MEM Medium and mixed, after which P3000 reagent was added and mixed again. The diluted DNA was then combined with the diluted Lipofectamine™ 3000 reagent and incubated for 20 min at room temperature. The resulting DNA–lipid complexes were applied to the cells. After 24 h, the medium was replaced with DMEM (high glucose) or other specified treatments.

### 2.10. Transient Transfection with ST6GAL1 siRNA

ST6GAL1 suppression was carried out in HDF cells using Lipofectamine™ RNAiMAX reagent (13778150, Invitrogen, Carlsbad, CA, USA) and the following siRNA-2 sequences: sense 5′→3′ CUCCCAACUAGGCAGAGAAAUTT; antisense 5′→3′ AUUUCUCUGCCUAGUUGGGAGTT. In brief, cells were plated to achieve 70–90% confluence at the time of transfection. Lipofectamine™ RNAiMAX was diluted in Opti-MEM Medium (31985070, Gibco, Waltham, MA, USA) and gently mixed. The siRNA was diluted separately in Opti-MEM Medium and mixed. The diluted siRNA was then combined with the diluted Lipofectamine™ RNAiMAX reagent and incubated for 20 min at room temperature. The resulting siRNA–lipid complexes were added to the cells for 24 h. Later, the medium was replaced with DMEM or other treatments, as specified.

### 2.11. T-Skin Culture and Treatment

The reconstructed human full-thickness skin model (T-Skin™) was obtained from EPISKIN (Lyon, France). The T-Skin™ three-dimensional in vitro reconstructed full-thickness skin model features a dermal layer composed of human fibroblasts and a well-stratified epidermal layer formed by differentiated normal human keratinocytes. Upon arrival, the T-Skin™ models were transferred to 6-well culture plates containing maintenance medium (SkinEthic™, EPISKIN, Lyon, France) and incubated at 37 °C with 5% CO_2_ for 24 h. After incubation, the T-Skin™ was exposed to UVA irradiation at a dose of 12 J/cm^2^ (365 nm) for 750 s using a UVA crosslinker (UCL-3500L, Luyor, Shanghai, China). After three consecutive days of irradiation, tissues were harvested for histological and immunohistochemical analysis 48 h after the final exposure to allow for the manifestation of the photoaged phenotype.

### 2.12. Tissue Paraffin Embedding

After irradiation, the central area of the T-skin™ (15 mm-diameter circle) was harvested and fixed in 4% paraformaldehyde solution (P0099, Beyotime, Shanghai, China). After fixation, the target regions were trimmed and placed into embedding cassettes with labels. Dehydration was performed using graded ethanol (1000874, XIHUA, Shanghai, China) (75%, 85%, 90%, 95%, and absolute ethanol twice), followed by ethanol–benzene, xylene (twice) (X821391, Macklin, Shanghai, China), and paraffin infiltration (three changes) at 65 °C. Tissues were then embedded in paraffin blocks using an embedding station (SWE-JBL5, Servicebio, Wuhan, China), cooled at −20 °C, and trimmed. Sections (4 µm) were cut with a microtome (PM-24, Servicebio, Wuhan, China), floated on 40 °C water, mounted onto slides, and baked at 60 °C. The slides were stored at room temperature for subsequent analyses.

### 2.13. Immunohistochemical Staining

Tissue sections were first dewaxed in xylene (X821391, Macklin, Shanghai, China) and rehydrated through a graded ethanol series (1000874, XIHUA, Shanghai, China). Heat-induced antigen retrieval was then performed using Tris-EDTA buffer (PR30002, Proteintech, Chicago, IL, USA). Following incubation with an endogenous peroxidase blocker (PV-6000, ZSGB-BIO, Beijing, China), the sections were blocked with 3% BSA for 1 h. Primary antibodies targeting P16 (10883-1-AP, Proteintech, Chicago, IL, USA), RAS (12063-1-AP, Proteintech, Chicago, IL, USA), ST6GAL1 (14355-1-AP, Proteintech, Chicago, IL, USA), ERK (66192-1-Ig, Proteintech, Chicago, IL, USA), MMP1 (10371-2-AP, Proteintech, Chicago, IL, USA), and MMP9 (10375-2-AP, Proteintech, Chicago, IL, USA) were each applied overnight at 4 °C at a 1:200 dilution. SNA (B-1305-2, Vector Laboratories, Newark, CA, USA) was applied for lectin histochemistry. Subsequently, the sections were incubated with an HRP-conjugated goat anti-mouse/rabbit IgG polymer secondary antibody (PV-6000, ZSGB-BIO, Beijing, China) for 20 min at 37 °C. Immunoreactivity was visualized using a DAB chromogenic kit (ZSGB-BIO, ZLI-9017), and nuclei were counterstained with hematoxylin (Beyotime, C0105). Images were captured under a light microscope (DMi8, Leica, Wetzlar, Germany).

### 2.14. Neuraminidase Activity Assay (4MU-NANA Assay)

Total cellular protein was extracted using CytoBuster™ Protein Extraction Reagent (71009, Novagen, Sacramento, CA, USA) according to the manufacturer’s instructions, and total protein concentrations were normalized to 1 mg/mL. Sialidase enzyme activity was measured using the fluorogenic substrate $2^{\prime }$-(4-methylumbelliferyl)-α-D-N-acetylneuraminic acid sodium salt hydrate (4MU-NANA; M8638, Sigma-Aldrich, St. Louis, MO, USA). Briefly, 100 μL of cell lysate (1 mg/mL) was mixed with 100 μL of 2 mM 4MU-NANA substrate solution prepared in phosphate-buffered saline (PBS, pH 7.4), yielding a final substrate concentration of 1 mM. The reaction mixture was incubated at 37 °C in the dark, and fluorescence intensity was recorded after 120 min using a microplate reader (Infinite F200, Tecan, Männedorf, Switzerland) at an excitation wavelength of 380 nm and an emission wavelength of 505 nm. Heat-denatured cell lysates served as negative controls. Background fluorescence from negative controls was subtracted from all readings, and relative sialidase activity was normalized to total protein content and calculated relative to non-irradiated control samples.

### 2.15. Statistical Analysis

Data were analyzed with GraphPad Prism (version 9.0; GraphPad Software Inc., San Diego, CA, USA). All data were first checked with the Shapiro–Wilk normality test. An unpaired Student’s *t*-test and one-way analysis of variance (ANOVA) were conducted to evaluate statistical differences between experimental groups. Tukey’s post hoc test was applied to adjust for multiple comparisons. Statistical significance is indicated as * *p* < 0.05, ** *p* < 0.01, *** *p* < 0.001, and **** *p* < 0.0001.

## 3. Results

### 3.1. Repeated UVA Exposures Induce Photoaging Phenotype in Human Dermal Fibroblasts

Sustained UVA exposure has been widely utilized to establish in vitro skin photoaging models in human dermal fibroblasts (HDFs), characterized by observable senescent morphology, increased β-galactosidase-positive cells, and reduced type I procollagen expression [16,17]. Building on these established methods, we developed a refined experimental protocol in which HDFs were subjected to UVA irradiation (5 J/cm^2^) once daily for three consecutive days (Figure S2A). To validate the photoaging phenotype, key senescence biomarkers were assessed. Markers associated with cell cycle arrest and DNA damage such as p16, p21, and γ-H2AX were elevated following UVA exposure, while collagen I expression was downregulated (Figure S2B). UVA also increased the proportion of senescent cells, as indicated by a rise in SA-β-galactosidase-positive HDFs (Figure S2C). Additionally, the expression of SASP-associated genes (*MMP1*, *MMP3*, *MMP9*, *MMP10*) was upregulated after repeated UVA irradiation (Figure S2D). Collectively, these results confirm that UVA-exposed HDFs recapitulate the hallmark features of skin senescence.

### 3.2. mRNA-Seq and Lectin-Array Analyses Reveal Altered Glycosylation in Photoaged HDFs

**Figure 1:**
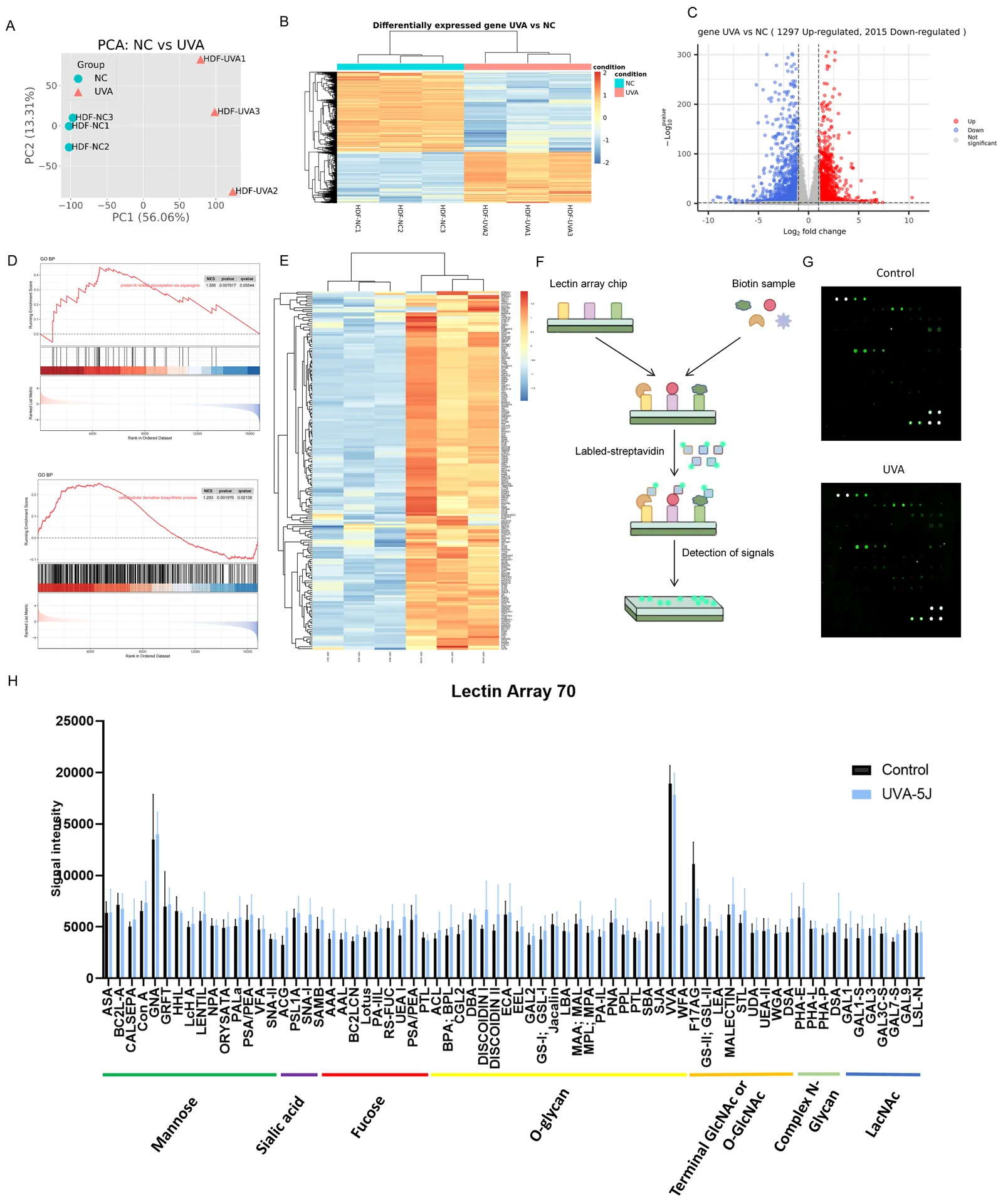
Transcriptomic profiling and lectin array reveal dysregulation of glycosylation in photoaged HDFs. (**A**) Principal Component Analysis (PCA) of HDF transcriptomes (*n* = 3). The first two principal components explained 56.1% and 13.3% of the total variance, respectively. (**B**) Hierarchical clustering heatmap of DEGs. (**C**) Volcano plot of differentially expressed genes (DEGs) in UVA-treated vs. NC HDFs. The vertical and horizontal dashed lines represent the log_2_FC thresholds (±1) and adjusted *p*-value threshold (0.05), respectively. (**D**) Gene Set Enrichment Analysis (GSEA) of differentially expressed genes. Enrichment plots for the biological processes “protein N-linked glycosylation via asparagine” and “carbohydrate derivative biosynthetic process” compare UVA-irradiated HDFs to non-photoaged controls. The Normalized Enrichment Score (NES) and nominal *p*-values indicate a significant positive enrichment of these pathways following UVA exposure. (**E**) Heatmap of the carbohydrate derivative biosynthetic pathway. Hierarchical clustering of core-enriched genes identifies a distinct transcriptomic signature in photoaged HDFs. Columns represent individual biological replicates, with the color scale indicating relative gene expression levels (red: upregulation; blue: downregulation). (**F**) Schematic representation of the label-based lectin microarray workflow. A systematic overview of the experimental pipeline, illustrating the isolation of membrane proteins from control and photoaged HDFs, followed by fluorescent labeling and incubation on a high-throughput array containing 70 immobilized lectins with diverse glycan specificities. (**G**) Representative fluorescence images of the lectin microarray. Comparative visualization of the raw fluorescence signal intensities between non-treated (control) and UVA-exposed HDF groups. The heightened signal intensity (in green) in the UVA-treated group reflects a global increase in lectin-binding glycoconjugates on the cell surface. (**H**) Systemic profiling of the photoaged dermal glycome. Differential analysis and hierarchical clustering of the lectin binding intensities reveal a shift in the glycomic landscape of photoaged HDFs. The data indicate a distinct fingerprint characterized by terminal modification and structural complexity, distinguishing the photoaged glycome from the quiescent control (N = 5).

We next conducted bulk mRNA sequencing for photoaged HDFs versus non-photoaged HDFs. The Principal Component Analysis (PCA) plot and hierarchical clustering revealed a good separation between control (NC) and UVA-treated HDFs (Figure 1A,B). As shown in Figure 1C, UVA exposure induced 3312 differentially expressed genes (DEGs) compared to the control group. To investigate the molecular pathways associated with photoaging, GSEA was performed (Figure 1D). The analysis revealed significant enrichment of the biological processes “protein N-linked glycosylation via asparagine” (NES = 1.556, q value = 0.05544) and “carbohydrate derivative biosynthetic process” (NES = 1.293, q value = 0.02138), indicating a strong association between photoaging in HDFs and dysregulation of these glycosylation- and carbohydrate-metabolism-related pathways. Consistent with the GSEA pathway enrichment, the gene expression heatmap for the “carbohydrate derivative biosynthetic process” revealed distinct clustering, with photoaged HDFs exhibiting a coordinated upregulation of core genes within this pathway compared to non-photoaged controls (Figure 1E).

To further elucidate the glycosylation profile change upon photoaging, we adopted a high-throughput lectin array that contained 70 immobilized lectins to detect glycan structures in photoaged and non-photoaged HDFs. A schematic plot is presented to reveal the label-based approach used in the experiment (Figure 1F). As indicated in Figure 1G, different intensity of fluorescence signals was observed in two groups, in which the signal intensity of UVA-exposed groups was higher than the non-treated groups. Our comprehensive lectin microarray profiling reveals that UVA-induced photoaging triggers a shift in the dermal glycome (Figure 1H). The most prominent changes are characterized by the hypersialylation (driven by Sambucus nigra lectin (SNA)) and hyper-fucosylation (driven by Ulex europaeus agglutinin (UEA) of the cell surface. Furthermore, we identified significant increases in Complex N-glycan branching by Datura stramonium agglutinin (DSA) and LacNAc presentation by galectin7-S lectin (GAL7-S), suggesting that photoaging promotes the elongation of glycan chains. In contrast, mannose-rich structures remained relatively stable. This high sialylation, high fucosylation, and increased overall N-glycan complexity signature provides a distinct glycomic fingerprint of photoaged tissue and suggests a transition toward a more ‘complex’ and ‘shielded’ carbohydrate profile on the cell surface.

### 3.3. UVA-Induced Photoaged Glycan Remodeling Is Driven by ST6GAL1-Mediated Sialylation

To specifically characterize the glycan modifications and pinpoint the exact structural shifts occurring following UVA exposure, we performed a lectin-binding assay utilizing six fundamental lectins with distinct specificities: Concanavalin A (ConA) for mannose, Peanut Agglutinin (PNA) for T-antigen, Dolichos Biflorus Agglutinin (DBA) for N-acetylgalactosamine, UEA I for α1,2-linked fucose residues, Sambucus nigra lectin (SNA) for α2,6-linked sialic acid, and Ricinus Communis Agglutinin (RCA120) for galactose and lactose.

**Figure 2:**
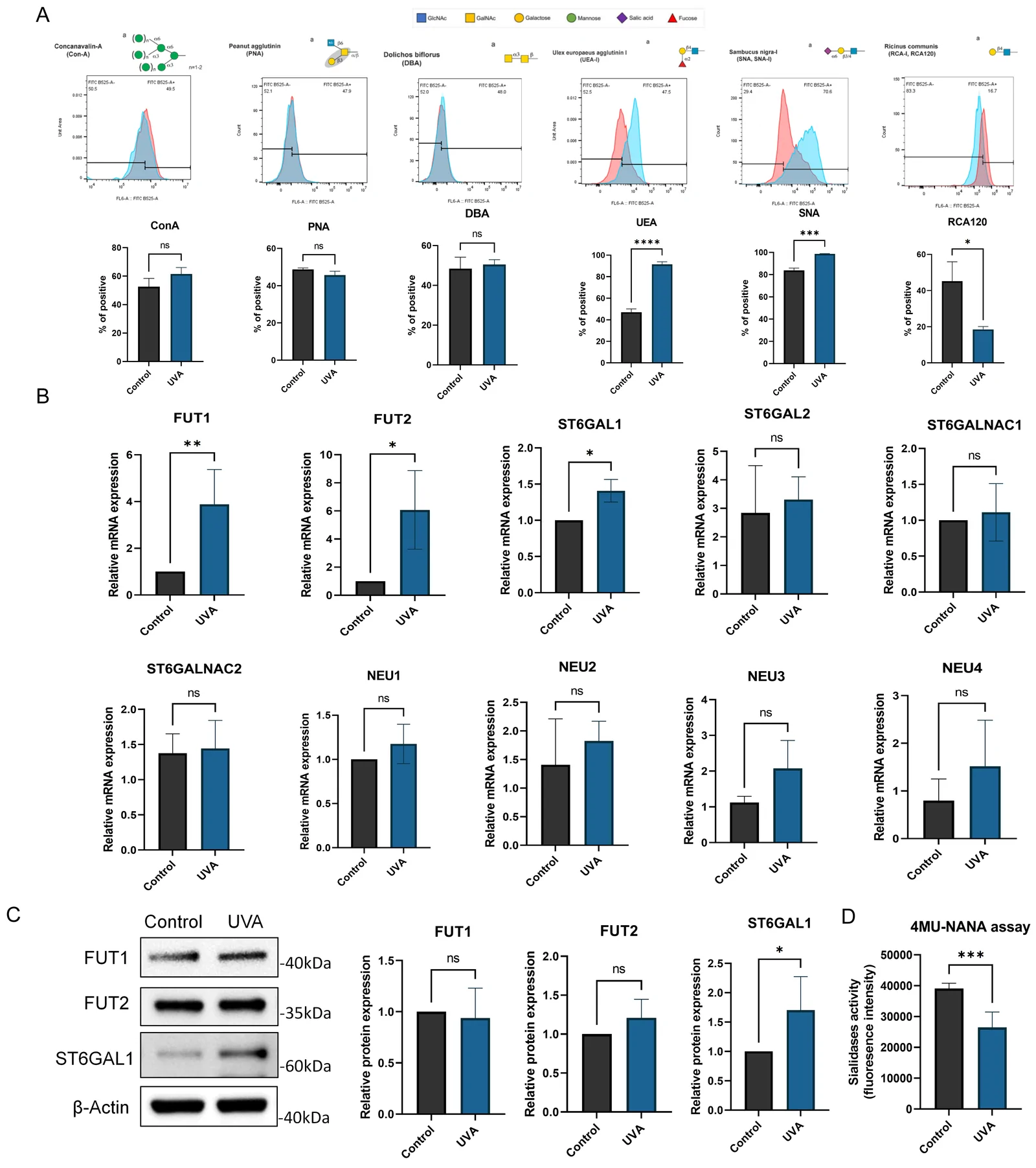
UVA-induced photoaged glycan remodeling is driven by ST6GAL1-mediated sialylation. (**A**) Flow cytometry analysis of lectin-binding assay using six fundamental lectins with distinct specificities (Con-A, PNA, DBA, UEAI, SNA and RCA120) for non-treated control (red) and UVA-exposed HDF groups (blue). (**B**) Quantitative RT-PCR analysis of fucosyltransferases, sialyltransferases and sialidases. Representative mRNA expression levels of *FUT1*, *FUT2*, *ST6GAL1*, *ST6GAL2*, *ST6GALNAC1*, *ST6GALNAC2*, *NEU1*, *NEU2*, *NEU3* and *NEU4* in control and UVA-irradiated HDFs. (**C**) Western blot analysis of FUT1, FUT2 and ST6GAL1 in control and UVA-irradiated HDFs. β-actin was utilized as an internal control. (**D**) Sialidase enzymatic activity in HDFs was quantified post-UVA exposure using 4MU-NANA fluorescence assay. Data are presented as mean ± SD (*n* = 4). * *p* < 0.05, ** *p* < 0.01, *** *p* < 0.001, **** *p* < 0.0001, ns: not significant vs. control (Student’s *t*-test).

As indicated in Figure 2A, we observed that only UEA I and SNA binding were significantly upregulated in photoaged human dermal fibroblasts (HDFs) compared to the control, whereas RCA120 binding was downregulated. These results imply that both α2,6-sialylation and α1,2-fucosylation represent the primary glycan alterations during the photoaging process. The observed decrease in RCA120 binding is consistent with these findings, as RCA120 typically detects unmasked terminal galactose residues; the addition of sialic acid or fucose often sterically inhibits RCA120 binding, effectively masking the underlying galactose.

To identify the specific enzymatic drivers responsible for these glycan changes, we examined the expression profiles of glycosyltransferases and glycosidases involved in α2,6-linked sialylation and α1,2-linked fucosylation (Figure 2B). Notably, the mRNA levels for the α1,2-fucosyltransferases *FUT1* and *FUT2*, as well as the α2,6-sialyltransferase *ST6GAL1*, were all significantly upregulated in the UVA-irradiated groups compared to controls. The expression of neuraminidases *NEU1*, *NEU2*, *NEU3,* and *NEU4* was unchanged upon UVA exposure.

We subsequently validated these changes at the protein level via Western blotting (Figure 2C). While multiple transcripts were elevated, we found that only the protein expression of ST6GAL1 was consistently increased in the UVA-induced photoaging model, whereas the protein levels of the tested fucosyltransferases remained unchanged. We also assessed global sialidase activity using a 4MU-NANA assay, which revealed a significant decrease in enzymatic activity within the UVA-irradiated group (Figure 2D). Consequently, based on this discordance between transcript and protein levels, all subsequent experiments were conducted with a focus on the functional role of ST6GAL1.

### 3.4. Sialyltransferase Inhibition Mitigates UVA-Induced Photoaging in HDFs

**Figure 3:**
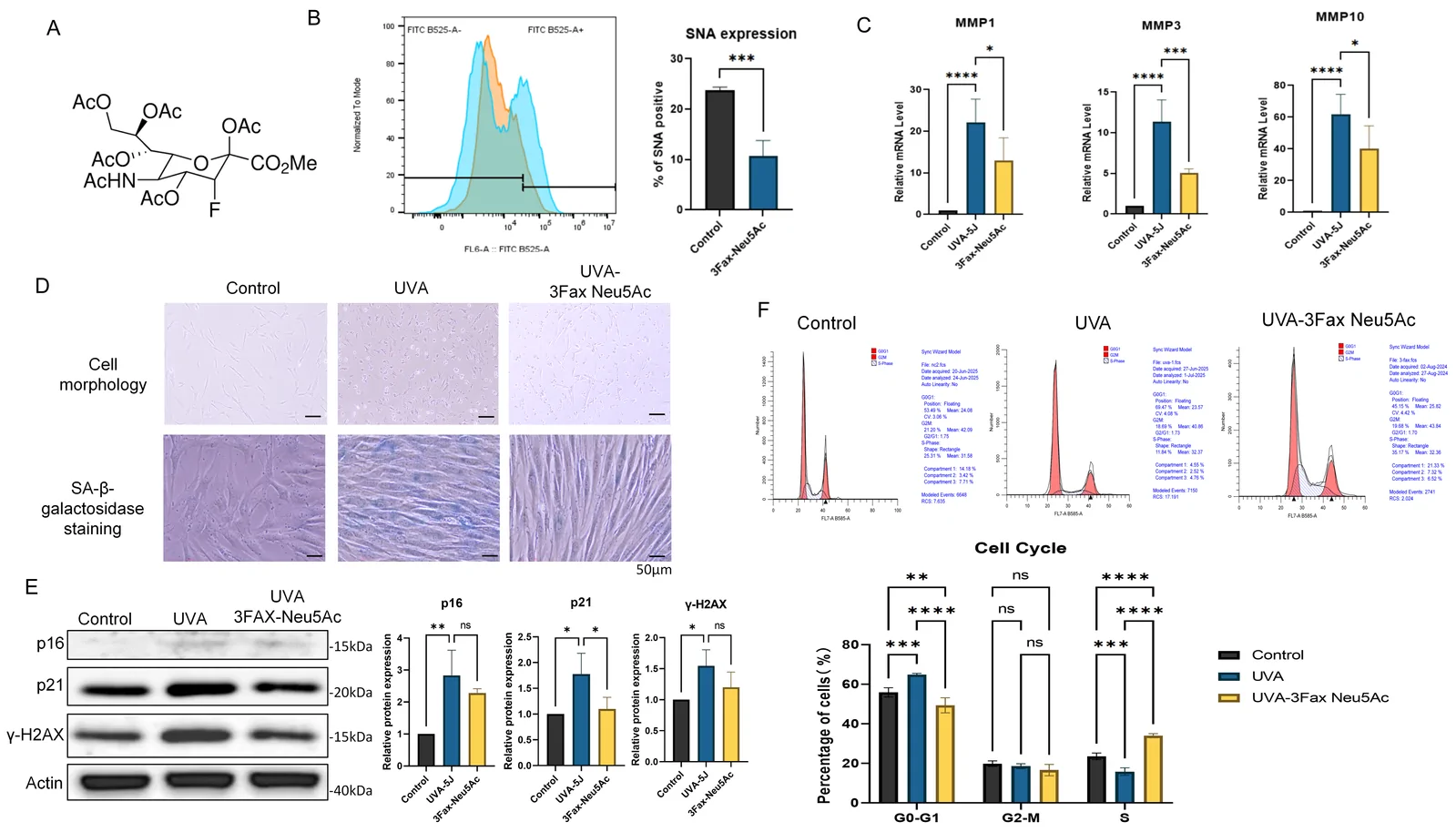
Pharmacological inhibition of sialyltransferase activity alleviates UVA-induced senescence. (**A**) Chemical structure of the sialyltransferase inhibitor 3Fax-Neu5Ac. (**B**) Flow cytometric analysis of SNA lectin binding in HDFs following treatment with 3Fax-Neu5Ac. (**C**) Relative mRNA expression levels of *MMP1*, *MMP3*, and *MMP10*. (**D**) Phenotypic assessment of HDF morphology and senescence. Representative bright-field images (top) showing cell morphology and SA-β-galactosidase staining (bottom) across three groups: control, UVA-irradiated (5 J/cm^2^), and UVA-irradiated with the sialyltransferase inhibitor 3Fax-Neu5Ac. (**E**) Protein expression levels of p16, p21, and γ-H2AX were analyzed via Western blot, with β-actin as the internal control. (**F**) Cell cycle distribution analysis by flow cytometry. Data are presented as mean ± SD (*n* = 4). * *p* < 0.05, ** *p* < 0.01, *** *p* < 0.001, **** *p* < 0.0001, ns: not significant (Student’s *t*-test for B and One-way ANOVA with Tukey’s post hoc test for the rest).

Given that α2,6-sialylation emerged as a hallmark of HDF photoaging and that ST6GAL1 expression appeared central to this remodeling, we sought to cross-validate these findings by pharmacologically modulating sialic acid metabolism. To directly inhibit sialylation, we treated photoaged HDFs with the peracetylated sialic acid analog and sialyltransferase inhibitor 3Fax-Neu5Ac (64 μM) (Figure 3A) [18]. Flow cytometric analysis of SNA binding confirmed that treatment effectively downregulated cell surface sialylation compared to untreated photoaged controls (Figure 3B).

We subsequently evaluated whether this inhibition could ameliorate the photoaged phenotype. Remarkably, treatment led to an observable reduction in SA-β-gal staining and suppressed the expression of key SASP components, including *MMP1*, *MMP3*, and *MMP10* (Figure 3C,D). Consistent with these observations, immunoblotting demonstrated a marked decline in the protein levels of the senescent markers p16, p21, and γ-H2AX following inhibitor treatment (Figure 3E). Cell cycle analysis also revealed that 3Fax-Neu5Ac treatment rescued the G0/G1 phase arrest induced by UVA exposure, which was characterized by concomitant recovery of the S-phase population (Figure 3F). Collectively, these data indicate that the inhibition of sialyltransferase activity effectively mitigates the photoaged phenotype in HDFs, suggesting a functional role for aberrant sialylation in driving cellular senescence.

### 3.5. ST6GAL1 Overexpression Induces a Photoaging-like Phenotype

**Figure 4:**
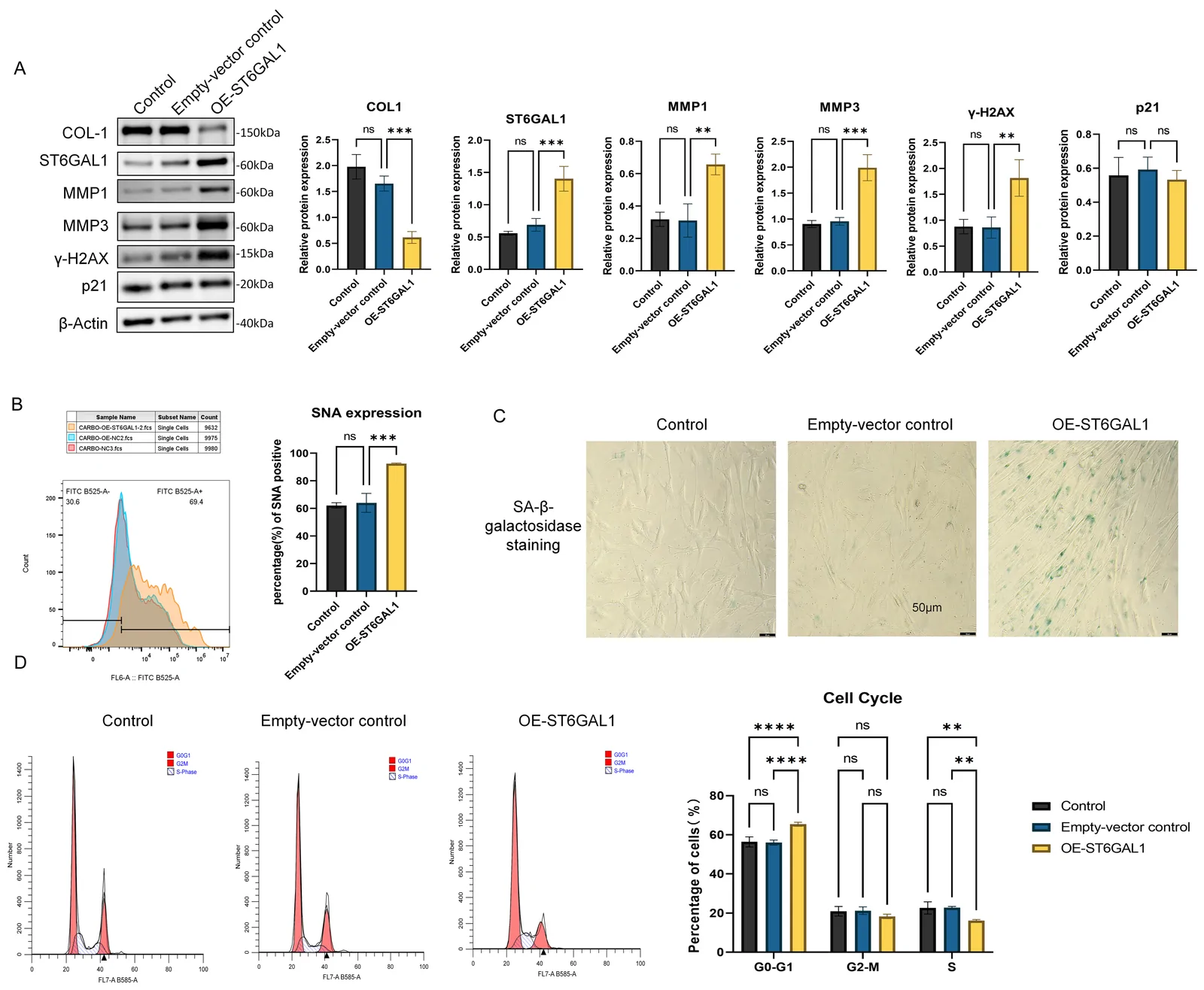
Overexpression of ST6GAL1 can mimic photoaged phenotype. (**A**) Western blot analysis of COL-1, ST6GAL1, MMP1, MMP3, γ-H2AX, and P21 in control HDFs, empty-vector control HDFs and ST6GAL1-OE HDFs. β-actin was utilized as an internal control. (**B**) Flow cytometry analysis for lectin binding of SNA in control HDFs, empty-vector control HDFs, and ST6GAL1-OE HDFs. (**C**) Detection of cellular senescence via SA-β-galactosidase staining in control HDFs, empty-vector control HDFs and ST6GAL1-OE HDFs. (**D**) Cell cycle analysis by flow cytometry for control HDFs, empty-vector control HDFs and ST6GAL1-OE HDFs. Data are presented as mean ± SD (*n* = 4). ** *p* < 0.01, *** *p* < 0.001, **** *p* < 0.0001, ns: not significant. One-way ANOVA with Tukey’s post hoc test.

To investigate the functional role of ST6GAL1 in driving photoaging, we established an overexpression (OE) model in HDFs via lipofection-mediated transfection. Immunoblotting revealed that ST6GAL1-OE HDFs exhibited a significant increase in the expression of senescence markers, including MMP1, MMP3, and γ-H2AX, alongside a concomitant reduction in COL1 protein levels compared to both untreated and empty-vector controls (Figure 4A). However, p21 was found to be unchanged across different treatments. Flow cytometric analysis confirmed that ST6GAL1-OE led to an induction of α2,6-sialylation, as evidenced by increased SNA binding (Figure 4B). Furthermore, SA-β-gal staining demonstrated a markedly higher proportion of positive cells in the ST6GAL1-OE group relative to controls (Figure 4C). Consistent with a senescent state, cell cycle analysis indicated that ST6GAL1-OE induced a significant arrest in the G0/G1 phase (Figure 4D). Collectively, these findings demonstrate that the overexpression of ST6GAL1 is sufficient to induce a photoaged phenotype in HDFs.

### 3.6. ST6GAL1 Drives Photoaging Through the Activation of the RAS-ERK-p16 Signaling Axis

**Figure 5:**
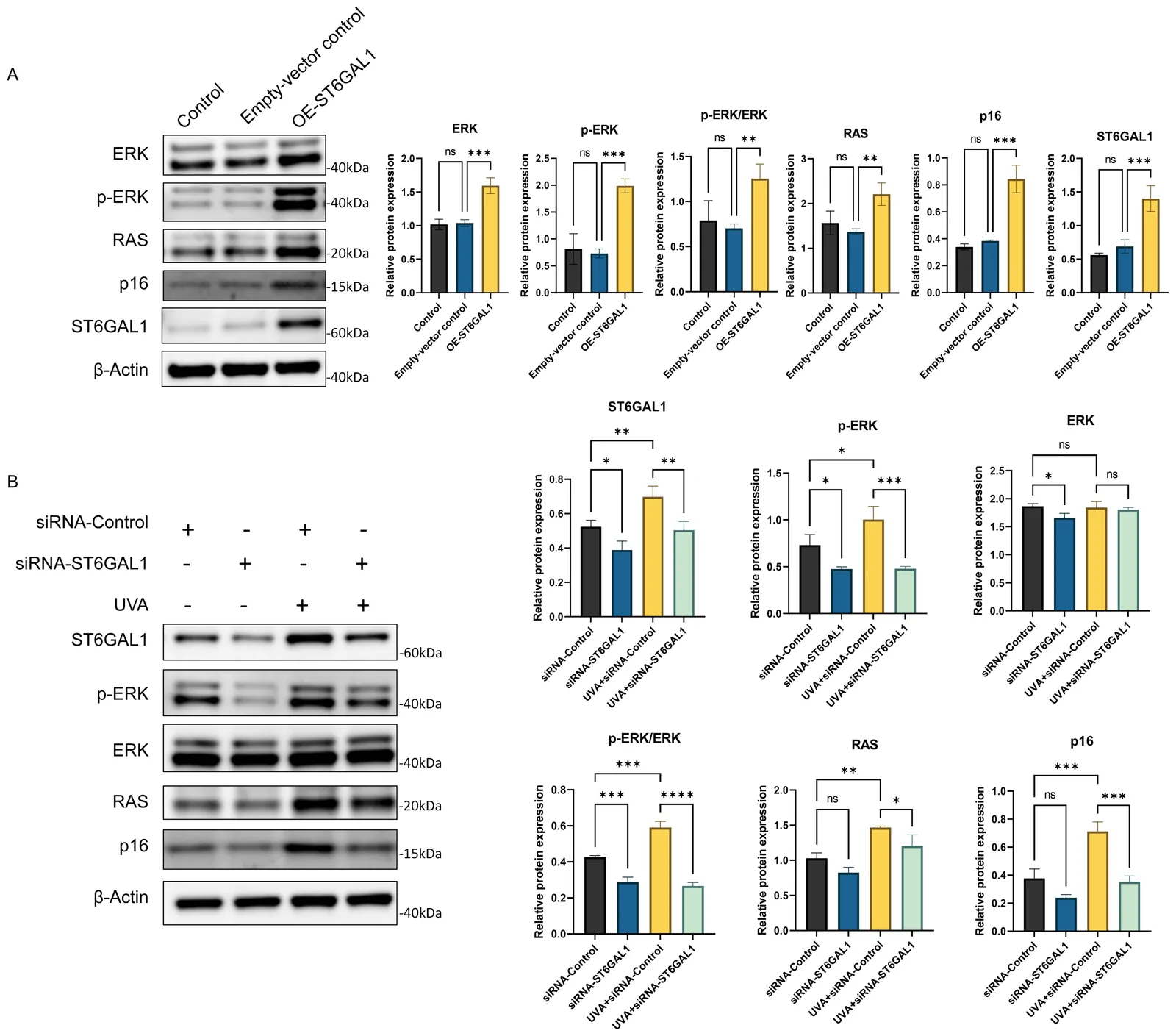
ST6GAL1 drives photoaging through the activation of the RAS-ERK-p16 signaling axis. (**A**) Western blot analysis of ERK, pERK, RAS, p16 and ST6GAL1 in control HDFs, empty-vector control HDFs and ST6GAL1-OE HDFs. β-actin was utilized as an internal control. (**B**) Western blot analysis of ERK, pERK, RAS, p16 and ST6GAL1 in siRNA-control HDFs, siRNA-ST6GAL1 HDFs, siRNA-control HDFs with UVA exposure and siRNA-ST6GAL1 HDFs with UVA exposure. β-actin was utilized as an internal control. Data are presented as mean ± SD (*n* = 4). * *p* < 0.05, ** *p* < 0.01, *** *p* < 0.001,**** *p* < 0.0001, ns: not significant. One-way ANOVA with Tukey’s post hoc test.

To elucidate the molecular mechanisms by which ST6GAL1 governs cellular senescence, we focused our investigation on the RAS-ERK signaling axis, as previous studies reported the association between RAS and ST6GAL1 [19] and identified RAS as a potent inducer of senescence-associated markers like p16 [20]. Consistent with this hypothesis, ST6GAL1-OE HDFs exhibited a marked upregulation of RAS, ERK, phosphorylated ERK (p-ERK), and the ratio of p-ERK/ERK compared to both untreated and empty-vector controls (Figure 5A), suggesting that the ST6GAL1-driven photoaging phenotype is mediated by this signaling pathway.

To further validate the necessity of ST6GAL1 in this process, we employed a loss-of-function approach using specific siRNAs (siRNA-1) to silence ST6GAL1, which effectively diminished both its protein (Figure S3A) and mRNA levels (Figure S3B). We then examined whether ST6GAL1 deficiency could suppress the RAS-ERK-p16 axis induced by photoaging. While UVA exposure significantly elevated ST6GAL1, RAS, ERK phosphorylation (p-ERK and p-ERK/ERK ratio), and p16 expression in empty-vector controls, ST6GAL1 depletion (UVA + siRNA-ST6GAL1) effectively reversed these effects. Specifically, knockdown of ST6GAL1 markedly inhibited UVA-induced RAS expression, suppressed ERK phosphorylation without altering total ERK levels, and attenuated p16 expression (Figure 5B). Collectively, these results confirm that ST6GAL1 is essential for UVA-induced photoaging and acts as a mediator of the RAS-ERK-p16 signaling pathway.

### 3.7. UVA-Induced Photoaging Triggers ST6GAL1-Mediated Sialylation and Senescence in a Reconstructed Human Full-Thickness Skin Model

**Figure 6:**
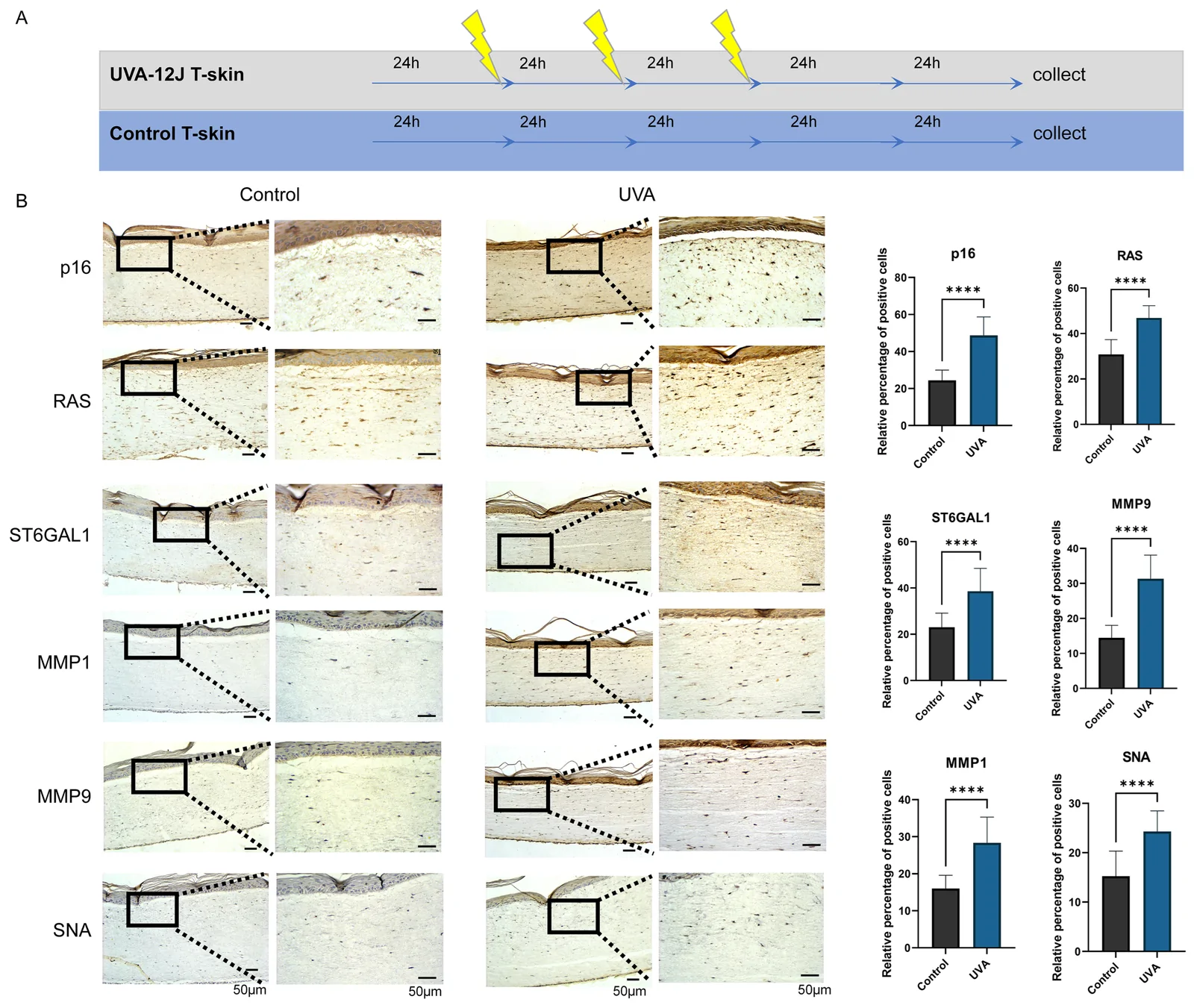
Validation of photoaging phenotype and ST6GAL1 in a 3D reconstructed human full-thickness skin model. (**A**) Schematic of the UVA irradiation protocol for 3D skin models. Full-thickness reconstructed human skin equivalents (T-skin™), containing both stratified keratinocytes and a fibroblast-populated collagen matrix, were subjected to repeated UVA (flash symbol) at a dose of 12 J/cm^2^ (365 nm) for three consecutive days. Tissues were harvested for histological and immunohistochemical analysis 48 h following the final exposure to allow for the manifestation of the photoaged phenotype. (**B**) Immunohistochemical (IHC) validation of photoaging signaling. Representative IHC images of T-skin cross-sections stained for p16, RAS, ST6GAL1, MMP1, MMP9 and SNA. Scale bar = 50 µm. Data are presented as mean ± SD. **** *p* < 0.0001 (Student’s *t*-test). *n* = 3.

To validate our findings in a physiologically relevant model that recapitulates the architectural complexity of human skin, we employed a reconstructed human full-thickness skin model (T-Skin™) composed of primary human fibroblasts and keratinocytes (Figure 6A). Histological assessment via H&E staining revealed that while control tissues maintained a well-defined, multi-layered stratified epidermis and a cohesive dermal compartment, repeated UVA irradiation (12 J/cm^2^ each day for a total of three doses) induced marked epidermal thinning and dermal disorganization (Figure S4A). Masson’s trichrome staining further corroborated UVA-induced structural decline, as evidenced by reduced collagen density and significantly fragmented fiber bundles.

Immunohistochemical (IHC) analysis demonstrated a robust increase in the senescence marker p16, alongside the matrix metalloproteinases MMP1 and MMP9, confirming the induction of a characteristic photoaged phenotype (Figure 6B). Crucially, both ST6GAL1 expression and SNA reactivity, as major indicators of α2,6-linked sialylation, were significantly elevated in UVA-exposed tissues in dermal compartments, reinforcing ST6GAL1 as a potential biomarker of cutaneous photoaging. Furthermore, increased staining for RAS was also observed in photoaged T-skin™, suggesting the induction of a signaling cascade. Collectively, these data demonstrate that UVA-induced photoaging in a 3D skin model is characterized by ECM degradation and a distinct shift in the sialylation landscape mediated by ST6GAL1.

## 4. Discussion

In this study, we identified the sialyltransferase ST6GAL1 and its mediated α2,6-sialylation as a previously unrecognized driver of UVA-induced fibroblast senescence. While the classical paradigm of skin photoaging has long centered on the immediate consequences of DNA damage and the cascade of ROS, the complex biochemical remodeling of the cell surface and secretory glycomics has remained largely underexplored. Through a combination of in vitro HDFs and a reconstructed human full-thickness skin model, our findings demonstrate that UVA exposure does not merely damage cellular components but actively recalibrates the enzymatic machinery of glycosylation, specifically through the upregulation of ST6GAL1. This discovery positions sialylation as a critical bridge between environmental UV stress and the stable, self-sustaining state of cellular senescence. By pinpointing ST6GAL1 as the enzymatic driver of this glycan shift, we provide a functional link showing how UVA stress can induce cell-surface glycan remodeling during photoaging. Consequently, ST6GAL1-mediated sialylation represents both a characteristic biomarker of dermal photoaging and a potential molecular target for interventions aimed at maintaining skin homeostasis.

Glycoconjugates facilitate a diverse array of biological processes, frequently serving as fine-tuning rheostats for cellular and molecular interactions [21]. Consequently, glycosylation profiles undergo profound remodeling under pathological conditions, especially in malignancy, inflammation, and the aging process [22,23]. Recent high-throughput analyses of large-scale cohorts have identified characteristic age-associated shifts in the N-glycome of serum and other biological fluids, suggesting that these glycan alterations are systemic hallmarks of biological aging [23,24]. Sialic acid, a nine-carbon monosaccharide typically situated at the terminal position of glycoprotein glycans, is a critical determinant of glycan structural stability, metabolic turnover, receptor binding and, thus, biological function [21]. They are typically conjugated to galactose or N-acetylgalactosamine through α2,3- or α2,6-linkages, or to other sialic acid residues via α2,8-linkages, generating diverse monomeric, oligomeric, and polymeric configurations. Due to its relatively strong electronegative properties, sialic acid plays a key role in numerous cellular processes and alterations in sialylation levels are believed to be functionally associated with both developmental and pathological conditions [25]. Sialylation change, specifically, was found to be related in various aging process. The sialic acid concentration alteration was found to be related to aging in muscle and testicular components [26,27]. *N*-glycomic analysis also revealed an increase in multi-branched and highly sialylated N-glycans observed in Japanese semisupercentanarians plasma proteins [28]. Consistent with our study, Oinam et al. demonstrated that increased in α2,6-sialylation expression was found in old epidermal stem cells, indicating impaired stem cell regenerative capacity and contributing to skin aging [14].

ST6GAL1, which is also referred to as β-galactoside alpha-2,6-sialyltransferase 1, is a type II membrane protein that transfers the α2,6-sialic acid monosaccharide to galactose-containing substrates [29]. It plays a critical role in cancers, aggressive diseases and stem cell pluripotency regulation [30,31,32]. Notably, the biological function of ST6GAL1 appears to be highly context-dependent; while it promotes aggressive phenotypes in several malignancies, its overexpression in hepatocellular carcinoma was reported to inhibit tumor metastasis by reducing cell migration and invasion [33]. In our study, we found that ST6GAL1 expression was significantly upregulated in response to UVA-mediated photoaging, leading to the hypersialylation of cell surface receptors in HDFs. We also observed a reduction in sialidase activity, which may also be a secondary contributor to α2,6-sialylation change. After treatment with the sialyltransferase inhibitor 3Fax-Neu5Ac in UVA-exposed HDFs, α2,6-sialylation expression was reduced and the photoaged phenotype was alleviated, although it affects multiple sialyltransferases and does not selectively establish ST6GAL1 dependence. In multiple myeloma cells, pretreatment with 3Fax-Neu5Ac altered cellular functions by interfering with cell-adhesion-mediated drug resistance [34]. We interpret that this enzymatic modification facilitates the capture of a stable senescence phenotype characterized by permanent cell cycle arrest, driven by persistent activation of the RAS-ERK-p16 signaling axis. Conversely, the experimental silencing of ST6GAL1 has been shown to ameliorate the senescent phenotype, suggesting that the sialylation state of the dermal glycome represents a reversible regulatory node in the photoaging process. The expression of ST6GAL1 is regulated by a complex network of transcription factors and epigenetic modifications. During UVA-induced photoaging, ROS are elicited which further trigger the activation of transcription factors like HNF1, which have also been shown to bind the ST6GAL1 promoter [35]. Indeed, the concept of “Glyco-redox” suggests that oxidative stress can trigger a redox switch that facilitates the cooperative functioning of the glycosylation machinery to modify the cell surface landscape in response to injury [36]; therefore, the activation of glycosyltransferases such as ST6GAL1 can be a plausible mechanism that requires further investigation.

The central hypothesis connecting ST6GAL1 to the senescence phenotype involves the hyperactivation of receptor tyrosine kinases and their downstream effectors [37]. In several models of cellular stress and transformation, ST6GAL1 has been shown to sialylate critical receptors such as the epidermal growth factor receptor (EGFR) and β-integrins [35]. The addition of α2,6-sialic acid to these receptors prevents their internalization and subsequent lysosomal degradation, thereby sustaining their half-life and prolonging the duration of signaling [37,38,39]. The RAS/RAF/MEK/ERK signaling pathway, which is further related to cell cycle regulation, is one of the most well-described pathways associated with ST6GAL1 activation [35]. Decades ago, ST6Gal1 was reported to be increased with overexpression of N-Ras in NIH 3T3 fibroblasts [40]. When Ras GTPase is recruited and activated, this further initiates the mitogen-activated protein kinase (MAPK) cascade, which triggers the phosphorylation of ERK1/2 that represents the primary conduit for the premature senescence signal involving the p53 and p16^INK4a^ [41]. The ST6GAL1-RAS-ERK-p16 signaling cascade we reported in this study provides a comprehensive explanation for how a change in the glycan landscape can dictate the terminal fate of dermal fibroblasts during photoaging.

The role of ST6GAL1 in senescence is supported by its correlation with multiple established biomarkers. In human mesenchymal stem cells (hMSCs), ST6GAL1 has been identified as part of a core set of genes that are differentially expressed during in vitro senescence, appearing alongside other markers of lysosomal dysfunction and metabolic reprogramming [42]. Moreover, the elevation of ST6GAL1 expression was also found in old epidermal stem cells, in which the overexpression of ST6GAL1 leads to compromised proliferation in epidermal stem cell culture [14]. The presence of ST6GAL1 in these profiles suggests that its role in aging is not limited to the dermis but may be a conserved feature of stromal cell senescence.

While this study establishes ST6GAL1-mediated α2,6-sialylation as a hallmark of UVA-induced photoaging, several mechanistic questions remain to be elucidated. A primary limitation of the current work is that we focused on the global shift in α2,6-sialylation rather than identifying the specific glycoprotein substrates of ST6GAL1. Future studies utilizing high-sensitivity sialoproteomics will be necessary to map the specific glycosylation sites and identify specific proteins that undergo these modifications to imply further functional relevance [43]. Although the global sialyltransferase inhibitor 3Fax-Neu5Ac was utilized to block sialylation, it lacks strict specificity for ST6GAL1 over other sialyltransferase isoforms. Our in vitro findings mainly focus on HDFs that rely on a single primary source; however, given the complexity of the skin microenvironment, it is imperative to investigate how ST6GAL1-driven sialylation facilitates crosstalk between fibroblasts, keratinocytes, and infiltrating immune cells. Future research should prioritize clinical validation in human skin biopsies with in vivo models to determine if ST6GAL1 expression or activity triggers a systemic pathogenic cascade in skin photoaging. Another limitation of the present study is that RAS involvement was evaluated based on total protein levels rather than direct GTP-bound RAS activation assays. Consequently, our findings support an association between ST6GAL1 expression and activation of the downstream ERK–p16 signaling pathway, though direct ST6GAL1-dependent RAS GTPase activation remains to be definitively established. Moreover, while we have characterized the sialylation landscape in UVA-induced photoaging, a comparative analysis with replicative senescence is warranted. Determining whether hypersialylation is a universal feature of aging or a stress-specific response to UVA radiation will be critical for developing targeted glyco-therapeutics that distinguish between intrinsic and extrinsic skin aging.

## 5. Conclusions

In conclusion, this study provides a comprehensive characterization of the altered glycomic landscape in skin photoaging, identifying ST6GAL1-mediated α2,6-sialylation as a central driver of dermal fibroblast senescence. By integrating high-throughput lectin profiling with functional molecular biology, we demonstrate that UVA radiation does not merely cause random macromolecular damage but initiates a coordinated enzymatic remodeling of the cell surface glycome.

Our data reveal a previously unrecognized hierarchical signaling cascade where ST6GAL1 acts as a critical regulator of the RAS-ERK-p16 axis. Our observation that ST6GAL1 overexpression mirrors key pathological aspects of photoaged HDFs, specifically the induction of cellular senescence and loss of ECM integrity, establishes a causal link between cell-surface glycan remodeling and the progression of extrinsic skin aging. Furthermore, the partial attenuation of the photoaged phenotype via ST6GAL1 silencing and sialyltransferase inhibition underscores the translational potential of these findings.

Ultimately, this research expands the current paradigm of photoaging beyond oxidative stress and DNA damage, placing enzymatic glycosylation at the forefront of cutaneous aging research. Targeting the ST6GAL1–sialylation axis may offer a novel strategy for developing glyco-geroprotective interventions to preserve dermal homeostasis under UV exposure.

## Figures and Tables

**Table 1:** Primer sequences used for qPCR analysis.

Gene	Forward Primer (5′→3′)	Reverse Primer (5′→3′)
*β-Actin*	CATGTACGTTGCTATCCAGGC	CTCCTTAATGTCACGCACGAT
*MMP1*	AAAATTACACGCCAGATTTGCC	GGTGTGACATTACTCCAGAGTTG
*MMP3*	AGTCTTCCAATCCTACTGTTGCT	TCCCCGTCACCTCCAATCC
*MMP9*	GGGACGCAGACATCGTCATC	TCGTCATCGTCGAAATGGGC
*MMP10*	TGCTCTGCCTATCCTCTGAGT	TCACATCCTTTTCGAGGTTGTAG
*FUT1*	CTTCCTGCTAGTCTGTGTCCT	ATTGGGGTAGACAGTCCAGGT
*FUT2*	TCCCCTGGCAGAACTACCA	GGTGAAGCGGACGTACTCC
*ST6GAL1*	AACTCTCAGTTGGTTACCACAGA	GGTGCAGCTTACGATAAGTCTT
*ST6GAL2*	AAGGGGAACGTCTCTTCCAAA	CTTGTTGGCGGTCAGGTAATC
*ST6GALNAC1*	AGAAAGGTCTCTACAGTCCCTG	TGTGTGTTGAGGGCATTGTTC
*ST6GALNAC2*	ACTTCCGTGGCCTGTTCAATC	GGCGATGACTTGGTGAGAGAG
*NEU1*	GGAGGCTGTAGGGTTTGGG	CACCAGACCGAAGTCGTTCT
*NEU2*	CCATGCCTACAGAATCCCTGC	CTCTGCGTGCTCATCCTTC
*NEU3*	AAGTGACAACATGCTCCTTCAA	TCTCCTCGTAGAACGCTTCTC
*NEU4*	GGCCACGGGATGACAGTTG	CAGGCGGATACCCATGTGTAG

## Data Availability

RNA sequencing data are deposited in GSE329475.

## Supplementary Materials

The following supporting information can be downloaded at: https://www.mdpi.com/article/10.3390/cells15161497/s1, Figure S1: The map of Lectin Array 70. Figure S2: Establishment and validation of the UVA-induced HDF photoaging model. Figure S3: Validation of siRNA-mediated knockdown of ST6GAL1 in HDF. Figure S4: UVA irradiation disrupted skin architecture and collagen integrity in a 3D T-skin model.
