# Supplementary material for: ST6GAL1 Is a Functional Regulator of UVA-Induced Photoaging in Human Dermal Fibroblasts
Source: Cells. 2026 Aug 20;15(16):1497. doi: 10.3390/cells15161497 (PMC13511385; doi:10.3390/cells15161497)
Supplement: Supplementary file 1 [file cells-15-01497-s001.zip › cells-4460272-supplementary.pdf]

Each lectin is spotted in duplicate horizonally

	A	B	C	D	E	F	G	H	I	J	K	L
1	POS1		POS2		NEG		NEG		AAA		AAL	
2	ACG		ACL		ASA		BanLec		BC2L-A		BC2LCN	
3	BPA		Calsepa		CGL2		CNL		Con A		DBA	
4	Discoidin I		Discoidin II		DSA		ECA		EEL		F17AG	
5	Gal1		Gal1-S		Gal2		Gal3		Gal3C-S		Gal7-S	
6	Gal9		GNA		GRFT		GS-I		GS-II		HHA	
7	Jacalin		LBA		LcH		LEA		Lentil		Lotus	
8	LSL-N		MAA		Malectin		MOA		MPL		NPA	
9	Orysata		PA-IIL		PA-IL		PALa		PHA-E		PHA-L	
10	PHA-P		PNA		PPL		PSA		PSL1a		PTL	
11	RS-Fuc		SAMB		SBA		SJA		SNA-I		SNA-II	
12	STL		UDA		UEA-I		UEA-II		VFA		VVA	
13	WFA		WGA		NEG		NEG		POS2		POS1	

Supplementary Figure S1. The map of Lectin Array 70.

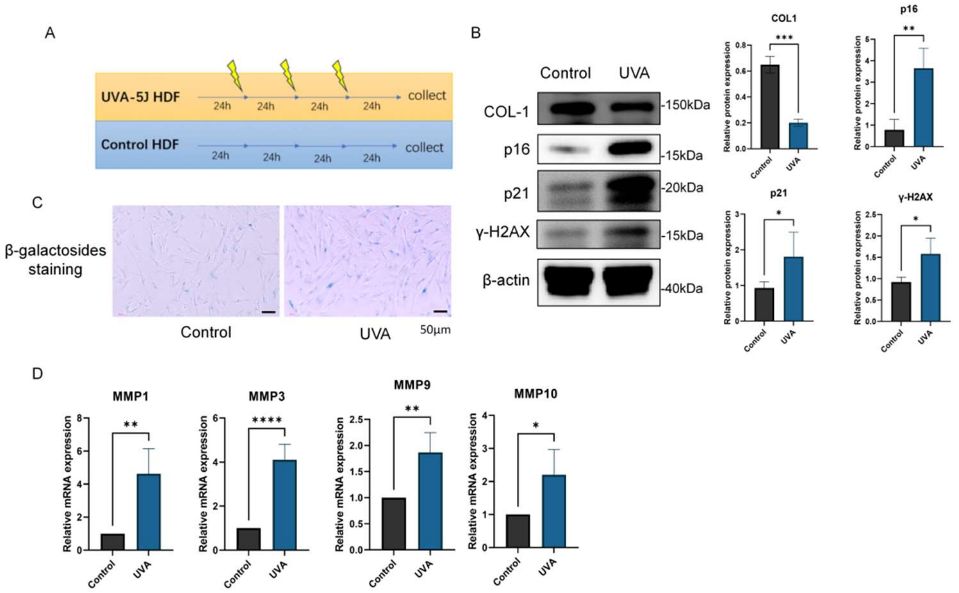

Supplementary Figure S2. Establishment and validation of the UVA-induced HDF photoaging model.

(A) Experimental schematic of the UVA irradiation protocol. HDFs were subjected to repeated UVA irradiation (5 J/cm^2 per dose) at 24-hour intervals for a total of three doses, with final cell collection performed 24 hours following the last treatment. Control HDFs were maintained under identical conditions without UVA exposure.

(B) Western blot analysis of senescence-associated markers. Representative protein expression levels of Type I Collagen (COL-1), p16, p21, and γ-H2AX in control and UVA-irradiated HDFs. β-actin was utilized as an internal control.

(C) Detection of cellular senescence via β-galactosides staining. Representative images of senescence-associated β-galactosidase (SA-β-gal) staining in control and UVA-treated HDFs.

(D) Quantitative RT-PCR analysis of the Senescence-Associated Secretory Phenotype (SASP). Relative mRNA expression levels of Matrix Metalloproteinases MMP1, MMP3, MMP9 and MMP10 in control and UVA-treated HDFs.(E) ROS level in control and UVA-treated HDFs.

Data are presented as mean ± SD (n = 4). *p < 0.05, **p < 0.01, ***p < 0.001 vs. Control (Student's t-test).

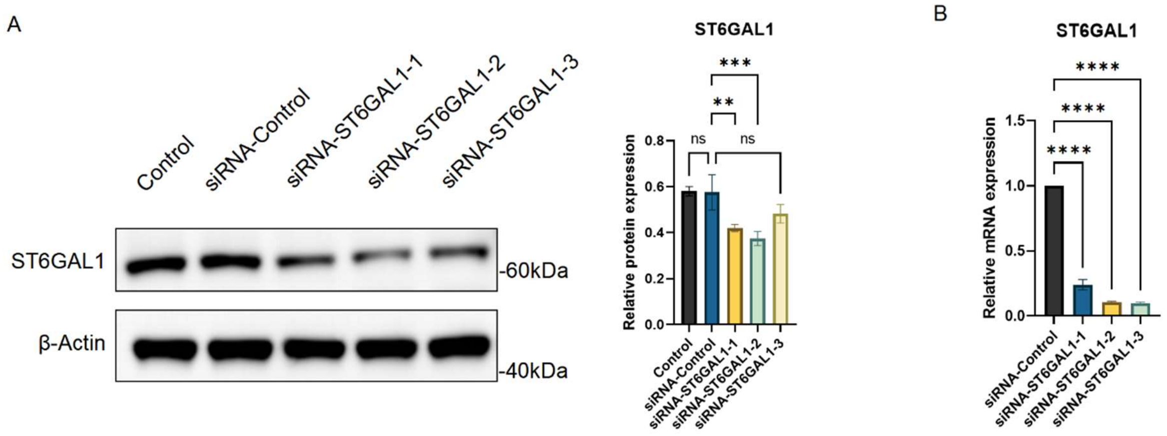

Supplementary Figure S3. Validation of siRNA-Mediated Knockdown of ST6GAL1 in HDF.

(A) Western blot analysis of ST6GAL1 protein expression. HDFs were transfected with three independent siRNA sequences targeting *ST6GAL1* (siRNA-ST6GAL1-1, -2, and -3) or a non-targeting control (siRNA-Control). Representative blots showing ST6GAL1 protein levels, with β-actin serving as the loading control.

(B) RT-qPCR analysis of ST6GAL1 mRNA levels. Relative mRNA expression of *ST6GAL1* was measured 48 hours post-transfection. All three siRNA sequences significantly reduced *ST6GAL1* transcript levels compared to the siRNA-Control.

Data are presented as mean ± SD (n = 4). *p < 0.05, **p < 0.01, ***p < 0.001, *****p < 0.0001 (One-way ANOVA with Tukey's post-hoc test).*

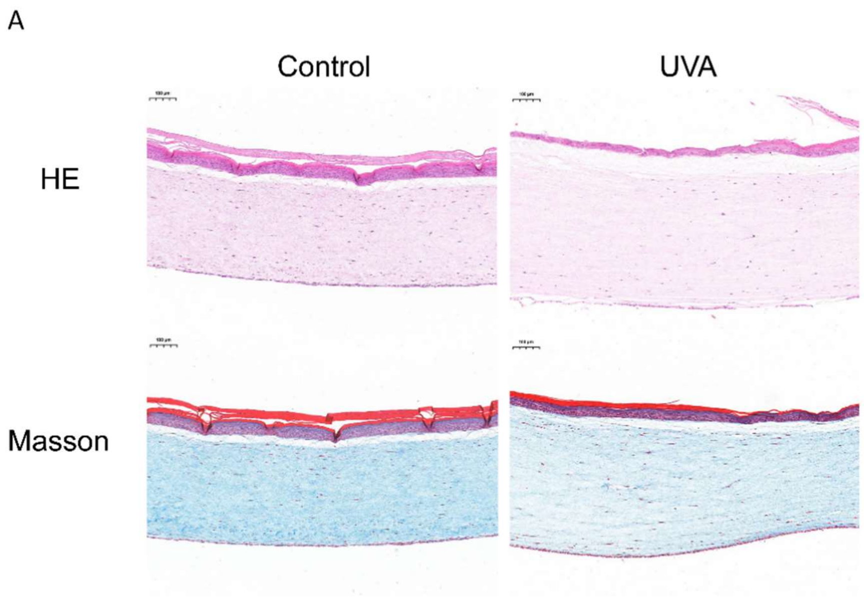

Supplementary Figure S4. **UVA Irradiation Disrupted Skin Architecture and Collagen Integrity in a 3D T-skin Model.**

(A) HE and Masson staining of cross-sections post-UVA exposure. Representative images show the structural differences between the non-irradiated control group (left) and the UVA-treated group (right). Scale bar=100μm.
